# Using Learning Techniques to Observe Elderly’s Behavior Changes over Time in Smart Home

**DOI:** 10.1007/978-3-030-51517-1_11

**Published:** 2020-05-31

**Authors:** Dorsaf Zekri, Thierry Delot, Mikael Desertot, Sylvain Lecomte, Marie Thilliez

**Affiliations:** 8 Digital Research Centre of Sfax, Sfax, Tunisia; 9 Institut Mines-Télécom, CNRS, Paris, France; 10 Université de Sherbrooke, Sherbrooke, QC Canada; 11 Digital Research Centre of Sfax, Sfax, Tunisia; 12 University of Sfax, Sfax, Tunisia; 13 Université Polytechnique Hauts-de-France, LAMIH UMR CNRS 8201, Hauts-de-France, France; 14 ReDCAD Laboratory, University of Sfax, B.P. 1173 Sfax, Tunisia

**Keywords:** Behavior change observation, Elderly people, Smart home, Activities of Daily Living

## Abstract

Smart environments and technology used for elder care, increases independent living time and cuts long-term care costs. A key requirement for these systems consists in detecting and informing about abnormal behavior in users’routines. In this paper, our objective is to automatically observe the elderly behavior over time and detect anomalies that may occur on the long term. Therefore, we propose a learning method to formalize a normal behavior pattern for each elderly people related to his Activities of Daily Living (ADL). We also adopt a temporal similarity score between activities that allows to detect behavior changes over time. In change behavior period we focus on each activity to detect anomalies. A use case with real datasets are promising.

## Introduction

With the growing elderly population, research in elderly living and well-being has been aimed toward medical analysis and supporting independent living of elderly people. Elderly people are often disabled by several interacting problems, such as loss of function and social and environmental factors. All these factors, separately or together, determine the elderly person’s level of independence and influence his/her quality of life.

In this context, most researchers aim to improve the living of elderly people with medical issues, such as diabetes and cognitive disabilities, by analyzing the behavior of residents within sensor-based environments. The progress of technology (wearable sensors, smart phones and other mobile devices, wireless communications, etc.) enables the development of effective solutions to help older people to live independently in their homes.

The smart home concept includes homes equipped with simple environmental sensors and more complex systems including audio, video and biometric systems. The raw information captured by the sensors can obviously not be shared as such with the medical staff or used directly to detect changes in behavior automatically. On the contrary, extracted knowledge could be used to enrich the information displayed to the medical staff and improve the precision of early detections. There is evidence that opportunistic home surveillance prevents in some cases hospitalization.

In this paper, we focus on the problem of learning from smart home sensor data describing elderly’s activities. Our objective in this work is to propose an approach to identify periods of time when behavior changes occur and detect anomalies in this period (e.g., the elderly sleep less and less every month). Our contributions in this paper can be summarized as follows.

1. We model a behavior pattern using training dataset, defined as the user’s usual activities in his/her daily routine.
2. We calculate a daily score by comparing activity patterns. This daily score variation provides a global vision of the behavior of the elderly person over a period of time.
3. We detect anomalies related to every activity in the period of behavioral deviation.

The rest of this paper is organized as follows. In Sect. 2, we discuss related works. In Sect. 3 we present our approach. In Sect. 4 we report the experimentation of our proposal on real datasets. In Sect. 5 we present our conclusions and some research directions.

## Related Works

With the use of smart homes, the daily activities and behavioral patterns of residents can now be monitored through sensors embedded within various areas in the home. This allows elderly people to be more independent while providing assistance to their family and caregivers. In this section, we describe some research works regarding the analysis of behavior and health monitoring for elderly people in the smart home context.

Works in [1] use anomaly detections on wearable sensors to provide an intelligent living environment for elderly residents. The detection of anomalies is based on several parameters: location, time, duration, type of activity and transitions between activities. The experiments provided consist in a semi-supervised learning approach.

 [2] and [3] study elderly residents diagnosed with dementia living independently in real home environments. They applied respectively neural networks and clustering algorithms to predict sensor activity. When an error is detected, timely audio or visual prompts are sent to the dementia patients.

Gjoreski et al. [4] have proposed a system to monitor users’daily activity by combining accelerometers with an electrocardiogram (ECG) sensor. Measured acceleration data can thus be analyzed in conjunction with the ECG signals to detect anomalies in the user’s behavior and heart-related problems.

Another detection strategy was proposed by Sprint et al. [5]. First, sensor data are labeled to correspond to activity “to sleep”. Features are then extracted and used as inputs to change detection algorithms such as RuLSIF, virtual classifier, and sw-PCAR to detect and analyze behavior changes that accompany health events. If the change is significant, change analysis is performed to explain the source of change. Use cases studied in this context concern older adults who experienced major health events, including cancer treatment and insomnia.

Anomaly detection systems for detecting abnormal behavior has been surveyed and reviewed in [6–8] implicitly rely of representation of the human activity in a spatiotemporal context highlighting various techniques/methods (classification, clustering, nearest neighbor, statistical).

Previous works describe existing research regarding the analysis of behavior and health monitoring from a smart home. A set of these works [1, 4] only use wearable sensors to monitor vital signs. Works in [2, 3, 5–8] consider home sensors to monitor daily activities but do not analyze all activities of the elderly person at the same time. For example, [5] studied the behavior change related to sleeping only. All the solutions mentioned previously have been developed to quickly detect and react as soon as possible when a sudden behavior change occurs, especially “the fall” of the monitored person. Our objective in this work is, not only to detect sudden changes, but also to analyze the possible evolution of the behavior over a long period of time.

## Our Approach

The overall objective of this study is to analyze the daily behavior of elderly people in their apartment through ambient sensors. In the following, we introduce our model to characterize the normal behavior pattern for elderly people. This normal behavior pattern can then be used to detect behavior changes over time by comparing the current behavioral data of an elderly with her/his usual behavior pattern.

### Activities and Daily Behavior Pattern

**Activities of Daily Living (ADLs)** is a term used by healthcare professionals to refer to the basic self-care tasks an individual does on a day-to-day basis. These fundamental activities are crucial for maintaining independence. They are used by health professionals as a way of measuring an individual’s functional status, especially for elderly people.

The importance of this issue has led to the development of numerous solutions that can monitor activities (e.g., [9]). Basic ADLs are self-care activities routinely performed which include, but are not limited to seven activities: sleeping, getting dressed, eating (three times per day), going to the toilet, hygiene activities (to take shower and/or bathing) and going outside.

Our notion of activity comprises two key criteria used also in [10] that are at the basis of our verification process:

1. Location: the specific place where an activity occurs, for example, “eating” takes place in the kitchen.
2. Time: the duration and occurring time of an activity. The user may perform a same activity at different times (e.g., going to the toilet) but some activities only occur at specific times of the day (e.g., eating breakfast). The start time and duration of each activity instance may be logged by the user, or better detected by an activity recognition system based on in-home sensors.

Let A = {$a_{1}; a_{2};...; a_{4}$} the set of activities labels. An activity pattern represents when and where an activity usually occurs. It is defined as a tuple:

$$\begin{aligned} P_{a} = (a_{i}, S_{a}(t), D_{a}(t)) \end{aligned}$$

where:

- $a_{i}\in A$ is an activity label
- $S_{a}(t)$ is a time interval representing the usual start time of activity $a_{i}$
- $D_{a}(t)$ is a time interval representing the usual duration of activity $a_{i}$

**The daily behavior pattern** involves several activity patterns. It defines order constraints on them and introduces eventual temporal delays. The daily behavior pattern describes how the user performs her/his activities at different times and models links between them. The daily behavior pattern is represented by a sequence of usual activities. It can be built from data derived from sensors in a smart home.

$$\begin{aligned} B = (P_{a1}, P_{a2}, P_{a3})\,\text {Where}\,P_{ai}\,\text {is an activity pattern} \end{aligned}$$

For each day of the week $D_{i}$ we built a behavior pattern $B_{i}$ which is a set of segments $P_{ai}$, where each segment $P_{ai}$ is a sequence of tuples ${a_{i}, S_{a}(t), D_{a}(t)}$ related to each activity. In this pattern we consider three activities: “sleeping”, “eating”, “taking a shower”, which occur at specific times of the day. “Going to the toilet” may occur at many times during the day. It will be studied separately as we will see later.

### Normal Behavior Pattern for the Elderly

The first step of any behavior anomaly detection system is to characterize the normal behavior, also called *routine behavior* or *regular behavior*, based on training data to model regularities in every individual activity. The normal behavior consists of the list of activities that a resident performs in her/his house, with time of the day and the duration. Thus, it captures the repetitive daily routines and deviations from the normal behavior may indicate changes of lifestyle or loss of capacity.

To build the routine behavior model, we follow the following steps:

1. We first use training data collected during the previous period which was treated as a baseline behavior period. We then follow an unsupervised learning approach: clustering to find point anomalies. To address this, we cluster instances of each activity based on start time and duration without considering the day of the week. For clustering, we use the DBSCAN algorithm [11] which is a density based clustering algorithm. The major advantage of DBSCAN, compared with other clustering algorithms like K-means, is that we do not need to specify how many clusters should be identified. After clustering, DBSCAN marks each point as belonging to a cluster or as noise(anomaly).
2. We eliminate point anomaly and we calculate the average start time and duration for each activity in training data.

The activity “going to the toilets” that occurs several times a day is treated separately. There are usually regular schedules for this activity. It is not essential to be very precise on the realization time of this activity. We then choose to study its frequency rather than the occurring time. Using the same training data to model regularities in the three studied activities, we calculate the frequency, per *n* hours, of the activity “going to the toilets”.

### Elderly’s Behavior Change Detection

Once computed, the normal behavior pattern can be used to detect anomalies by comparing the current behavioral data of an elderly with her/his normal behavior pattern. The basic idea of our behavioral deviation detection system is to estimate the similarity between both patterns using a score. We therefore consider three criteria for the activities: the time, duration and chronological order of the activities in the sequence.

**Behavior Modeling Using a Daily Activity Score:** Intuitively, a particular activity is similar to a pattern if its start time, duration and location are similar to the ones defined by the pattern. The similarity of the time and duration for each activity is estimated by a score.

The similarity score of an activity *a* in a day *d* is calculated by the formula (1). It is given as a percentage and represents the temporal intersection of the normal behavior pattern and one observed day pattern, for the same activity. We note that $S_{ad}$ is the start time of activity $a_{d}$, $D_{ad}$ is the duration of activity $a_{d}$ and $E_{ad}= S_{ad}+D_{ad}$ is the end time of activity $a_{d}$

1 $$\begin{aligned} Similarity\ score =\frac{(\inf (E_{an},E_{ad}),\sup (S_{an},S_{ad}))*100}{D_{an}} \end{aligned}$$

The similarity score for one day is the average of similarity scores for all activities occurring in this day.

The duration score is calculated by the formula (2). It is a percentage of the duration of an activity in an observed day compared to the duration of the same activity in the normal behavior pattern.

2 $$\begin{aligned} Duration\ score = \frac{D_{ad}*100}{D_{an}} \end{aligned}$$

The duration score for one day is the average of duration scores for all activities occurring on this day. The duration score can exceed 100% if the duration of an activity at the observed day is greater than the expected duration in the normal behavior pattern. This simply means that the elderly takes longer to achieve the activity which may be caused by a loss of autonomy if this is observed regularly.

**Fig. 1.**
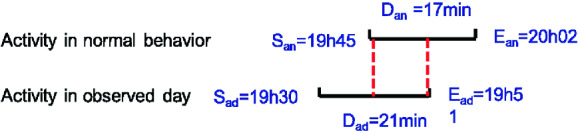
Illustration of the similarity and duration scores for the activity “eating lunch”

In the example presented in Fig. 1, the similarity score is 35.29%. It represents the temporal intersection for the activity “to eat lunch” in one observed day pattern compared to the same activity in the normal behavior pattern. The duration score (80.95% in our example) is the percentage of the duration of the activity “to eat lunch” in an observed day compared to the duration of the same activity in the normal behavior pattern.

The variation of these scores over time, represents the evolution of the elderly’s life pace. These scores thus give us an indication of the variation with an elderly’s usual behavior for a particular activity. A large decrease in these scores over a long period (from a few days to a few weeks) may be an initial signal of decline and should generate a notification to the caregivers or the family members.

For regular activities, occurring several times a day, we compare the frequency between a routine day and an observed day. By simply plotting the daily score along time, it is possible to identify certain days with unusual activities (i.e., with lower scores), or trends of evolution that indicate deviations from the previous activity routine.

A subsequent work detailed in Sect. 3.3 consists in studying in details the activities related to the behavior change period to discover the deviation cause(s).

**Anomaly Detection in Behavior Change Period:** In this section, we investigate the accuracy of anomaly detection: per day and per hour of the day, in the behavior change period. Activities in this period has been mapped and compared with normal life pattern.

In the domain of at-home activities, anomalies can be classified as point, collective and contextual anomalies. The *point anomaly* [12–14] considers each activity independently and decides whether it is normal or not with respect to the normal behavior. The *collective anomaly* [15] considers groups of activity instances together to determine whether the group is normal or not. The *contextual anomaly* [16, 17] considers activities under a particular context (e.g., day of week, person under medication, etc.). In our work, we focus on detecting *point anomaly* (i.e., missing activity or activity with an unusually long/short duration).

For the activity “going to the toilet” which occurs several times per day, we model and compare frequencies between a routine day and the observed day in the behavior change period. A frequency is provided by the normal behavior pattern. This will be illustrated in the following section.

## Use Case

In this section, we present in 4.1 the dataset used as use case. We detail in 4.2 the learning steps for building the normal behavior pattern. By plotting the daily score along time in 4.3, we exhibit the period when the score changes over time and then identify daily anomalous activities found in the behavior change period.

### Dataset

The dataset used for our analysis is provided by Washington State University’s CASAS program^1^ [18]. CASAS (Center for Advanced Studies in Adaptive Systems) aims to provide aid to residents using smart home technology. They therefore collect and use real-time data from sensors to analyze and monitor residents’health and behavior to improve future smart home living.

We use one public data set (named HH120) [18] which was used in other works like [19]. It includes one unique subject, covering a total of 63 days. All data used in this paper was handled in an anonymized way. The set of activities includes “sleeping”, “eating meals”, “taking a shower”, “going outside” and “going to the toilet”. The data sets do not provide any medical information. For training the normal behavior model, we use the first month while the rest of the available data is used to test the effectiveness of our proposals.

### Learning for Building the Normal Behavior Pattern

**Fig. 2.**
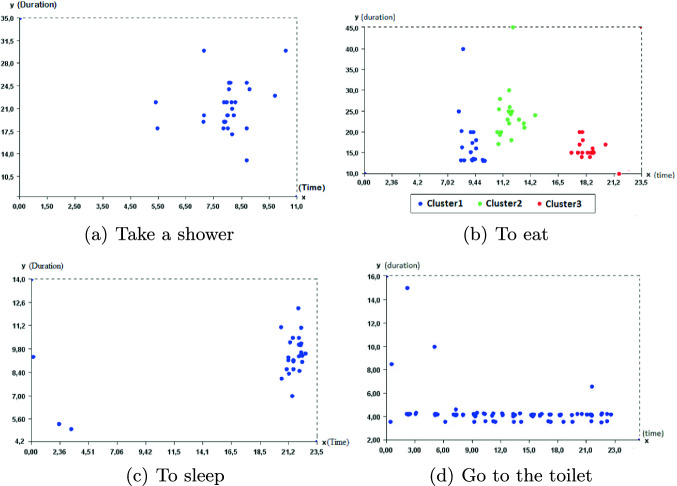
DBSCAN Clustering for detecting anomalies

For building the normal behavior pattern from the training data set, we follow the different learning steps presented in the Sect. 3.2. We then apply the DBSCAN algorithm on the training dataset for clustering and eliminate activities out of the identified clusters and marked as noise. DBSCAN has two parameters; one is *min_pts* which is the minimum number of points in a cluster, and the other is *Eps* which is the maximum distance between two data points for them to be considered in the same cluster. While learning, data out of *Eps* would be considered out of clusters so marked anomalous. Trained results are depicted in Fig. 2.

Figure 2(b) illustrate 3 clusters which represent 3 daily meals. The elderly person is habituated to have her breakfast at 9:44 AM for maximum 25 min. Figure 2(b) shows that this person can have her breakfast for 40 min which is abnormal behavior depicted by the point outside the middle cluster.

**Fig. 3.**
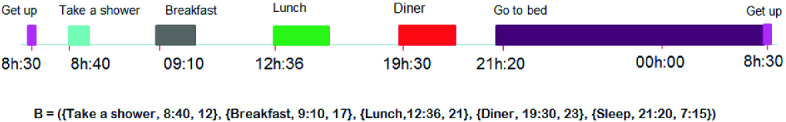
Normal behavior pattern

We then eliminate point anomalies and calculate, for every activity in the training data set representing one month of collected data, the average start time and average duration. Figure 3 illustrates the daily behavioral model thus generated using the previous learning step.

### Behavior Change Period and Anomalous Activities

**Fig. 4.**
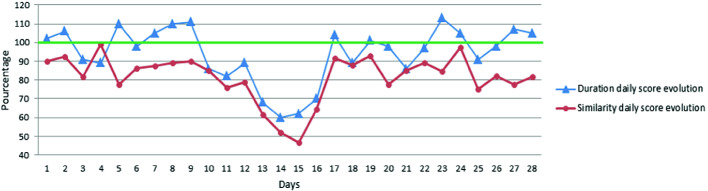
Daily scores evolution

In the first stage of our experiments, we computed the daily scores introduced in Sect. 3.3. By plotting scores, we can observe the behavior evolution day by day to follow the evolution of elderly’s life pace. Thus, it is possible to identify trends in the daily evolution scores as shown in Fig. 4 where we can observe a decrease compared to the previous routine activity.

**Fig. 5.**
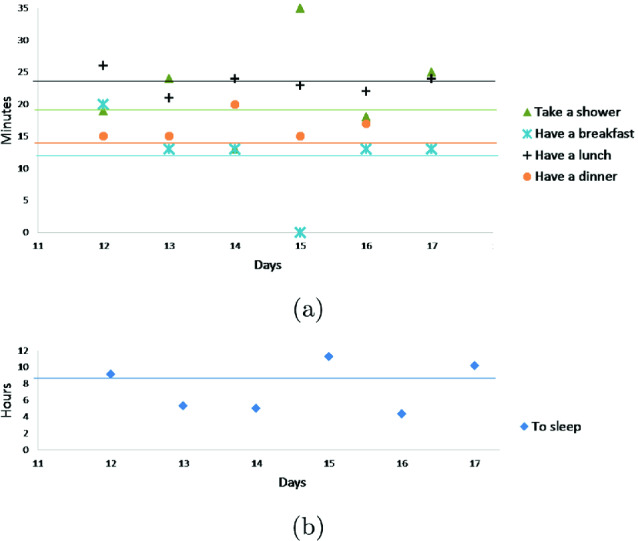
(a) Activities durations in observed days (b) sleeping duration

**Fig. 6.**
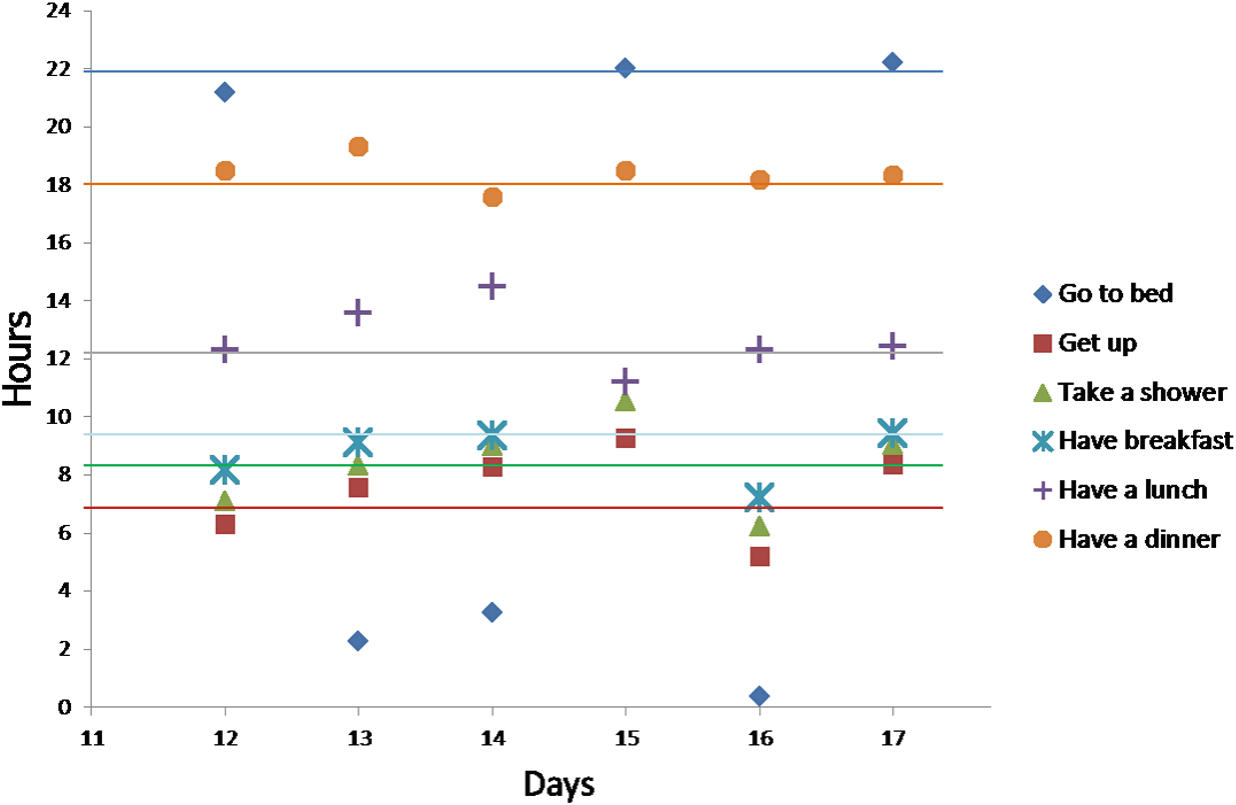
Activities start time in observed days

In the second stage of our experiment, we focus on the deviation period (days with decreasing/increasing scores) to detect point anomalies due to a missing activity or activities with unusually long/short durations. To do this, we plot in Figs. 5 and 6 duration and start time respectively for 3 activities (to sleep, to eat (breakfast, lunch, dinner) and take a shower). In these figures, the average start time and the average duration in normal behavior pattern are represented for each activity by an horizontal line.

At days 13 and 14, Figs. 5 and 6 reveal unusual sleep times, shorter than usual, as well as later times to go to bed (2:00 AM and 4:00 AM). The results also indicate that day 15 is a day with unusual activity because the elderly skipped a lunch. At the same day, the elderly performs more times than usual the activity “taking a shower” and “sleeping”. During these 3 days we detect 2 types of anomaly: point anomaly due to missing activity and activities in unusually long/short durations.

**Fig. 7.**
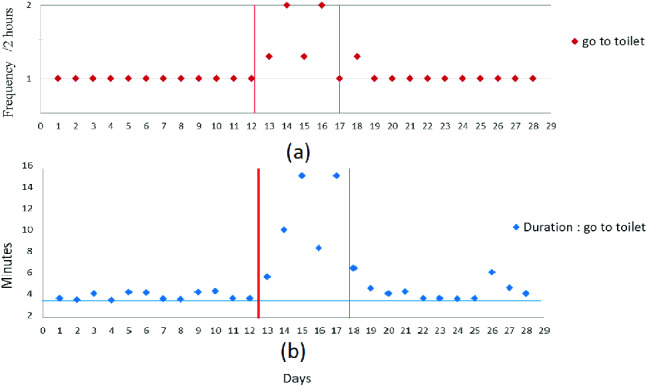
(a) Frequency “go to the toilet” (b) Duration “go to the toilet”

As mentioned previously, the activity “going to the toilet” that occurs several times a day is treated separately. As for the other activities, anomalies related to duration are eliminated using DBSCAN as illustrated in Fig. 2. To analyze the elderly’s behavior, we focus both on the frequency per 2 h and the duration. Figure 7 shows that both these parameters increase in the deviation period compared to the normal behavior (represented with the horizontal line).

All these anomalies may be a signal of sickness (urinary tract infection, gastrointestinal problem, etc.) so our system may send an alert message to inform remote caregivers.

## Conclusion

In this article, we presented our research work to detect behavioral changes in the elderly’s usual behavior. Our solution relies on the construction of a behavior model. Thanks to this scheme and based on the detection of anomalies, we are able to detect changes in the elderly behavior, not only sudden changes such as a fall or a temporary illness, but also changes over time. For example, the elderly sleep less and less every month, which can be worrying and cause many problems. In the future, we would like to integrate other activities into our model, such as “going outside”, which are an important aspect for characterizing the elderly’s health. This information should also be coupled with contextual elements such as weather conditions. Other information on health conditions can also be used to refine our detection of behavioral changes. Finally, when our system detects changes in behavior, it must make a decision on the appropriate solution: for example, if it is a sudden and persistent change, it may be an emergency solution and if it is a degradation of the subject’s daily routine, a visit from the caregiver would be necessary. It is difficult to make the “best” decision so we plan at this point to talk to healthcare professionals to configure our decision support system.

## Footnotes

1 http://casas.wsu.edu/.
